# Supplementary material for: An Efficient Way to Screen Inhibitors of Energy-Coupling Factor (ECF) Transporters in a Bacterial Uptake Assay
Source: Int J Mol Sci. 2022 Feb 27;23(5):2637. doi: 10.3390/ijms23052637 (PMC8910649; doi:10.3390/ijms23052637)
Supplement: Supplementary file 1 [file ijms-23-02637-s001.zip › ijms-1600130-SI.pdf]

International Journal of
Molecular Sciences

MDPI

Supplementary Materials

An efficient way to screen inhibitors of energy-coupling factor (ECF) transporters in a bacterial uptake assay

Spyridon Bousis [1,2,3], Steffen Winkler [1], Jörg Haupenthal [1], Francesco Fulco [1], Eleonora Diamanti [1] and Anna K. H. Hirsch [1,2,3*]

[1] Helmholtz Institute for Pharmaceutical Research (HIPS) – Helmholtz Centre for Infection Research (HZI), Campus Building E 8.1, D-66123, Saarbrücken, Germany;

[2] Stratingh Institute for Chemistry, University of Groningen, Nijenborgh 7, NL-9747 AG Groningen, the Netherlands.

[3] Department of Pharmacy, Saarland University, Campus Building E8.1, 66123 Saarbrücken, Germany.

*Corresponding author

Prof. A. K. H. Hirsch

Helmholtz Institute for Pharmaceutical Research Saarland (HIPS) – Helmholtz Centre for Infection Research (HZI)

Department of Drug Design and Optimization

Campus Building E8.1, 66123 Saarbrücken, Germany

e-mail: anna.hirsch@helmholtz-hips.de

Table of Contents

1. General information 2
2. Synthesis and characterization of compounds 4
Synthetic schemes 4
General procedure 5
3. Biochemical assays 21
4. Antibacterial testing 23
5. Sequence conservation 23
6. References 26

1. General information

Chemicals, Materials and Methods

NMR experiments were run on a Bruker Ultrashield plus 500 (500 MHz) spectrometer. Spectra were acquired at 300 K or at 500 MHz, using deuterated dimethylsulfoxide (DMSO–d_6) as solvent. Chemical shifts for ^{1}H and ^{13}C spectra were recorded in parts per million (ppm) using the residual non-deuterated solvent as the internal standard (for DMSO–d_6: 2.50 ppm, ^{1}H; 39.52 ppm, ^{13}C). Coupling constants (*J*) are given in Hertz (Hz). Data are reported as follows: chemical shift, multiplicity (s = singlet, d =doublet, t = triplet, m = multiplet, br = broad and combinations of these) coupling constants and integration. Flash chromatography was performed using the automated flash chromatography system CombiFlash Rf+ (Teledyne Isco, Lincoln, NE, USA) equipped with RediSepRf silica columns (Axel Semrau, Sprockhövel Germany). TLC was performed with aluminium-backed silica TLC plates (Macherey-Nagel MN ALUGRAM Sheets SIL G/UV 254 20 x 20cm 818133) with a suitable solvent system and was visualized using UV fluorescence (254 & 366 nm). All reactions were carried out in oven-dried glassware under an atmosphere of argon. Anhydrous DMF was purchased from Aldrich and used directly.

Mass spectrometry was performed on a SpectraSystems-MSQ LCMS system (Thermo Fisher, Dreieich, Germany) using a Hypersil Gold column, 150 x 3 mm, 5 µm. At a flow rate of 700 µL/min, the gradient of H_2O (0.1% FA) and ACN (0.1% FA) starting from 30% ACN and then increased to 95% over 12 min. The mass spectrum was measured in positive and negative mode in a range from 100–600 m/z. The UV spectrum was recorded at 254 nm. High-resolution mass spectra (HR-MS) were recorded with a ThermoScientific system where a Dionex Ultimate 3000 RSLC was coupled to a Q Exactive Focus mass spectrometer with an electrospray ion (ESI) source. An Acquity UPLC® BEH C8, 150 x 2.1 mm, 1.7 µm column equipped with a VanGuard Pre-Column BEH C8, 5 x 2.1 mm, 1.7 µm (Waters, Germany) was used for separation. At a flow rate of 250 µL/min, the gradient of (A) H_2O + 0.1% FA and (B) ACN + 0.1% FA was held at 10% B for 1 min and then increased to 95% B over 4 min. It was held there for 1.2 min before the gradient was decreased to 10% B over 0.3 min where it was held for 1 min. The mass spectrum was measured in positive mode in a range from 120–1000 m/z. UV spectrum was recorded at 254 nm. The compounds which did not give a good ionization at the Q Exactive, have been measured on a Dionex Ultimate 3000 RSLC system using a BEH C18, 100 x 2.1 mm, 1.7 µm dp column (Waters, Germany). Separation of 1 µl sample was achieved by a linear gradient from (A) H_2O + 0.1% FA to (B) ACN + 0.1% FA at a flow rate of 600 µL/min and 45 °C. The gradient was initiated by a 0.5 min isocratic step at 5% B, followed by an increase to 95% B in 18 min to end up with a 2 min step at 95% B before re-equilibration under the initial conditions. UV spectra were recorded by a DAD in the range from 200 to 600 nm. The LC flow was split to 75 µL/min before entering

the maXis 4G hr-ToF mass spectrometer (Bruker Daltonics, Germany) using the Apollo ESI source. Mass spectra were acquired in centroid mode ranging from 150–2500 m/z at a 2 Hz scan rate.

Abbreviations Acetonitrile (ACN), ammonium hydroxide (NH_4OH), boron trifluoride diethyl etherate ($BF_3{\cdot}Et_2O$), chloroform ($CHCl_3$), dichloromethane (DCM), dimethylformamide (DMF), dimethylsulfoxide (DMSO), ethyl acetate (EtOAc), formic acid (FA), hydrochloric acid (HCl), methanol (CH_3OH), sodium borohydride ($NaBH_4$), sodium chloride (NaCl), sodium triacetoxyborohydride $NaBH(OAc)_3$, potassium carbonate (K_2CO_3), potassium chloride (KCl), potassium hydrogen phosphate (K_2HPO_4) sodium sulfate (Na_2SO_4). Other abbreviations used are: aqueous (aq.), hours (h), minutes (min), room temperature (rt), on (overnight), saturated (sat.).

2. Synthesis and characterization of compounds

Synthetic schemes

The compounds **3**, **23**−**26** and **27** were prepared following a reductive amination reaction between the respective aldehydes **28**−**30** and the anilines **31**−**33**. Sodium triacetoxy boronhydride was used as the reductive reagent. To improve the yields, a 2-fold excess of amines was used.

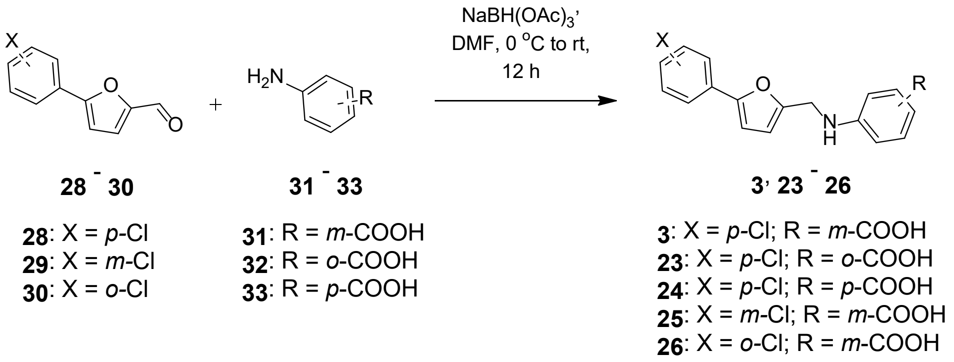

Figure S1. Preparation of the compounds **3** and **23**–**26** by using a reductive amination reaction.

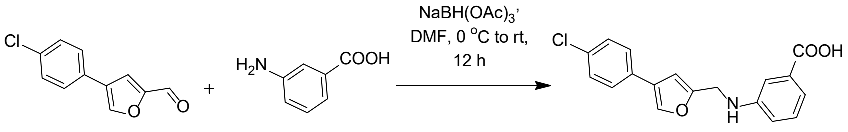

Figure S2. Preparation of compound **27** by using a reductive amination reaction.

General procedure

General procedure (GP1): reductive amination

To a stirred solution of carbaldehyde (1 eq) and aminobenzoic acid (2 eq) in dry DMF (0.2 M), was added at 0 °C portionwise sodium triacetoxyborohydride (2 eq) under nitrogen. The reaction mixture was stirred at 0 °C for 30 min and leave it stirring at rt for 12 hours. The reaction mixture was quenched slowly under ice with the addition of water. The crude product was extracted with EtOAc (3x). The combined organic layers were dried over Na_2SO_4, filtered, concentrated *in vacuo* and purified by flash chromatography.

3-(((5-(4-Chlorophenyl)furan-2-yl)methyl)amino)benzoic acid (**3**):

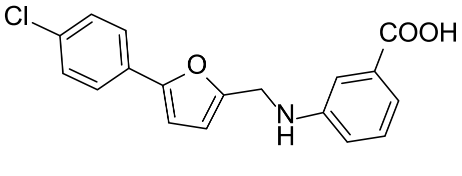

According to the GP1, using 5-(4-chlorophenyl)furan-2-carbaldehyde (0.100 g, 0.484 mmol), 3-aminobenzoic acid (0.133 g, 0.968 mmol) and sodium triacetoxyborohydride (0.205 g, 0.968 mmol) in DMF (2.5 mL) to give, after purification by column chromatography (hexane/EtOAc 7:3) **3** as yellow powder (0.120 g, 76 %). ^{1}H NMR (500 MHz, DMSO−d_6) δ 12.67 (s, 1H), 7.71 – 7.65 (m, 2H), 7.49 – 7.44 (m, 2H), 7.32 – 7.28 (m, 1H), 7.22 – 7.13 (m, 2H), 6.91 (dd, J = 8.1, 2.3, 2H), 6.46 (t, J = 6.0, 1H), 6.42 (d, J = 3.3, 1H), 4.35 (d, J = 5.9, 2H). ^{13}C NMR (126 MHz, DMSO) δ 168.3, 154.0, 151.4, 148.8, 132.0, 131.8, 129.7, 129.4, 129.3,

125.3, 117.6, 116.9, 113.5, 109.9, 107.7 (1 peak missing due do overlapping with DMSO). HR-MS (ESI) calcd for $C_{18}H_{13}ClNO_3$ [M–H]$^-$: 326.06622, found: 326.05902.

2-(((5-(4-Chlorophenyl)furan-2-yl)methyl)amino)benzoic acid (**23**):

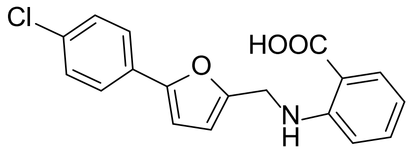

According to GP1, using 5-(4-chlorophenyl)furan-2-carbaldehyde (0.100 g, 0.484 mmol), 2-aminobenzoic acid (0.133 g, 0.968 mmol) and sodium triacetoxyborohydride (0.205 g, 0.968 mmol) in DMF (2.5 mL) to give, after purification by column chromatography (hexane/EtOAc 7:3) **23** as yellow powder (0.127 g, 80 %). ^{1}H NMR (300 MHz, DMSO−d_6) δ 12.70 (s, 1H), 8.24 (s, 1H), 7.81 (dd, J = 7.9, 1.6, 1H), 7.71 – 7.63 (m, 2H), 7.51 – 7.42 (m, 2H), 7.38 (ddd, J = 8.6, 7.1, 1.7, 1H), 6.96 – 6.86 (m, 2H), 6.65 – 6.56 (m, 1H), 6.45 (d, J = 3.3, 1H), 4.53 (s, 2H). ^{13}C NMR (75 MHz, DMSO) δ 169.9, 152.9, 151.2, 150.3, 134.4, 131.6, 131.6, 129.1, 128.9, 124.8, 114.9, 111.68, 110.6, 109.5, 107.4 (1 peak missing due do overlapping with DMSO). HR-MS (ESI) calcd for $C_{18}H_{13}ClNO_3$ [M–H]$^-$: 326.06622, found: 326.05890.

4-(((5-(4-Chlorophenyl)furan-2-yl)methyl)amino)benzoic acid (**24**):

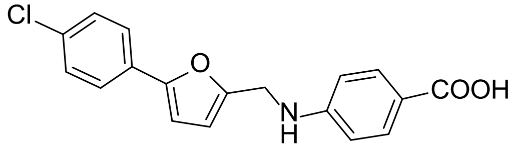

According to the GP1, using 5-(4-chlorophenyl)furan-2-carbaldehyde (0.100 g, 0.484 mmol), 4-aminobenzoic acid (0.133 g, 0.968 mmol) and sodium triacetoxyborohydride (0.205 g, 0.968 mmol) in DMF (2.5 mL) to give, after purification by column chromatography (hexane/EtOAc 7:3) **24** as yellow powder (0.1 g, 63 %). ^{1}H NMR (500 MHz, DMSO−*d₆*) δ 12.04 (s, 1H), 7.68 (dd, *J* = 8.4, 6.1, 4H), 7.47 (d, *J* = 8.4, 2H), 6.96 (t, *J* = 5.9, 1H), 6.93 (d, *J* = 3.3, 1H), 6.70 (d, *J* = 8.4, 2H), 6.45 (d, *J* = 3.3, 1H), 4.38 (d, *J* = 5.9, 2H). ^{13}C NMR (126 MHz, DMSO) δ 167.4, 153.0, 152.0, 151.1, 131.6, 131.0, 129.2, 128.9, 124.8, 117.6, 111.3, 109.7, 107.3 (1 peak missing due do overlapping with DMSO). HR-MS (ESI) calcd for $C_{18}H_{13}ClNO_3$ [*M*–H]$^{-}$: 326.06622, found: 326.37753.

3-(((5-(3-Chlorophenyl)furan-2-yl)methyl)amino)benzoic acid (**25**):

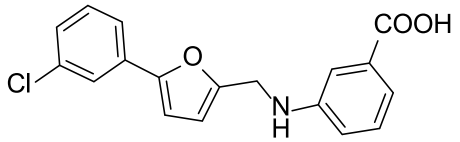

According to the GP1, using 5-(3-chlorophenyl)furan-2-carbaldehyde (0.200 g, 0.968 mmol), 3-aminobenzoic acid (0.266 g, 1.936 mmol) and sodium triacetoxyborohydride (0.41 g, 1.963 mmol) in DMF (4.8 mL) to give, after purification by column chromatography (hexane/EtOAc 7:3) **25** as yellow powder (0.21 g 67 %). ^{1}H NMR (500 MHz, DMSO−*d₆*) δ 12.67 (s, 1H), 7.70 (t, *J* = 1.9, 1H), 7.62 (dt, *J* = 7.8, 1.3, 1H), 7.42 (t, *J* = 7.9, 1H), 7.31 (ddd, *J* = 10.7, 2.4, 1.3, 2H), 7.22 – 7.13 (m, 2H), 7.00 (d, *J* = 3.3, 1H), 6.91 (ddd, *J* = 7.9, 2.5, 1.3, 1H), 6.46 (t, *J* = 6.1, 1H), 6.42 (d, *J* = 3.3, 1H), 4.35 (d, *J* = 6.0, 2H). ^{13}C NMR (126 MHz, DMSO) δ

168.3, 154.4, 150.9, 148.8, 134.2, 132.7, 131.8, 131.29, 129.4, 127.4, 123.2, 122.1, 117.7, 117.0, 113.5, 109.9, 108.5 (1 peak missing due do overlapping with DMSO). HR-MS (ESI) calcd for $C_{18}H_{13}ClNO_3$ [*M*–H]$^-$: 326.06622, found: 326.37817.

3-(((5-(2-Chlorophenyl)furan-2-yl)methyl)amino)benzoic acid (**26**):

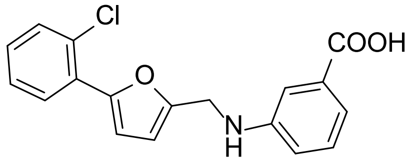

According to the GP1, using 5-(2-chlorophenyl)furan-2-carbaldehyde (0.100 g, 0.484 mmol), 3-aminobenzoic acid (0.133 g, 0.968 mmol) and sodium triacetoxyborohydride (0.205 g, 0.968 mmol) in DMF (2.5 mL) to give, after purification by column chromatography (hexane/EtOAc 7:3) **26** as yellow powder (0.132 g, 83 %). ^{1}H NMR (500 MHz, DMSO−d_6) δ 12.67 (s, 1H), 7.83 (dd, J = 7.9, 1.7, 1H), 7.53 (dd, J = 8.0, 1.3, 1H), 7.41 (td, J = 7.6, 1.3, 1H), 7.33 – 7.28 (m, 2H), 7.23 – 7.13 (m, 2H), 7.06 (d, J = 3.3, 1H), 6.92 (ddd, J = 7.9, 2.6, 1.2, 1H), 6.47 (dd, J = 7.2, 4.7, 2H), 4.37 (d, J = 6.0, 2H). ^{13}C NMR (126 MHz, DMSO) δ 168.3, 154.1, 148.8, 148.7, 131.8, 131.2, 129.4, 129.1, 129.1, 128.9, 128.0, 127.9, 117.7, 117.0, 113.5, 112.3, 109.7, 40.5, 40.3, 40.2, 40.0, 39.8, 39.6, 39.5. HR-MS (ESI) calcd for $C_{18}H_{13}ClNO_3$ [*M*–H]$^-$: 326.06622, found: 326.05750.

3-(((4-(4-Chlorophenyl)furan-2-yl)methyl)amino)benzoic acid (**27**):

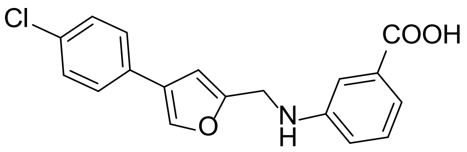

According to the GP1, using 4-(4-chlorophenyl)furan-2-carbaldehyde (0.8 g, 0.387 mmol), 3-aminobenzoic acid (0.106 g, 0.774 mmol) and sodium triacetoxyborohydride (0.165 g, 0.774 mmol) in DMF (2 mL) to give, after purification by column chromatography (hexane/EtOAc 6:4) **27** as yellow powder (0.67 g, 53 %). ^{1}H NMR (500 MHz, DMSO−d_6) δ 12.64 (s, 1H), 8.14 (d, J = 1.0, 1H), 7.58 (d, J = 8.5, 2H), 7.41 (d, J = 8.5, 2H), 7.27 – 7.22 (m, 1H), 7.22 – 7.11 (m, 2H), 6.88 (ddd, J = 8.0, 2.6, 1.2, 1H), 6.77 (s, 1H), 6.43 (t, J = 6.0, 1H), 4.30 (d, J = 5.9, 2H). ^{13}C NMR (126 MHz, DMSO) δ 168.3, 155.1, 148.8, 139.4, 131.7, 131.4, 129.4, 129.2, 127.5, 125.7, 117.6, 116.7, 113.5, 106.1 (2 peaks missing due do overlapping). HR-MS (ESI) calcd for $C_{18}H_{13}ClNO_3$ [M–H]$^-$: 326.06622, found: 326.37808.

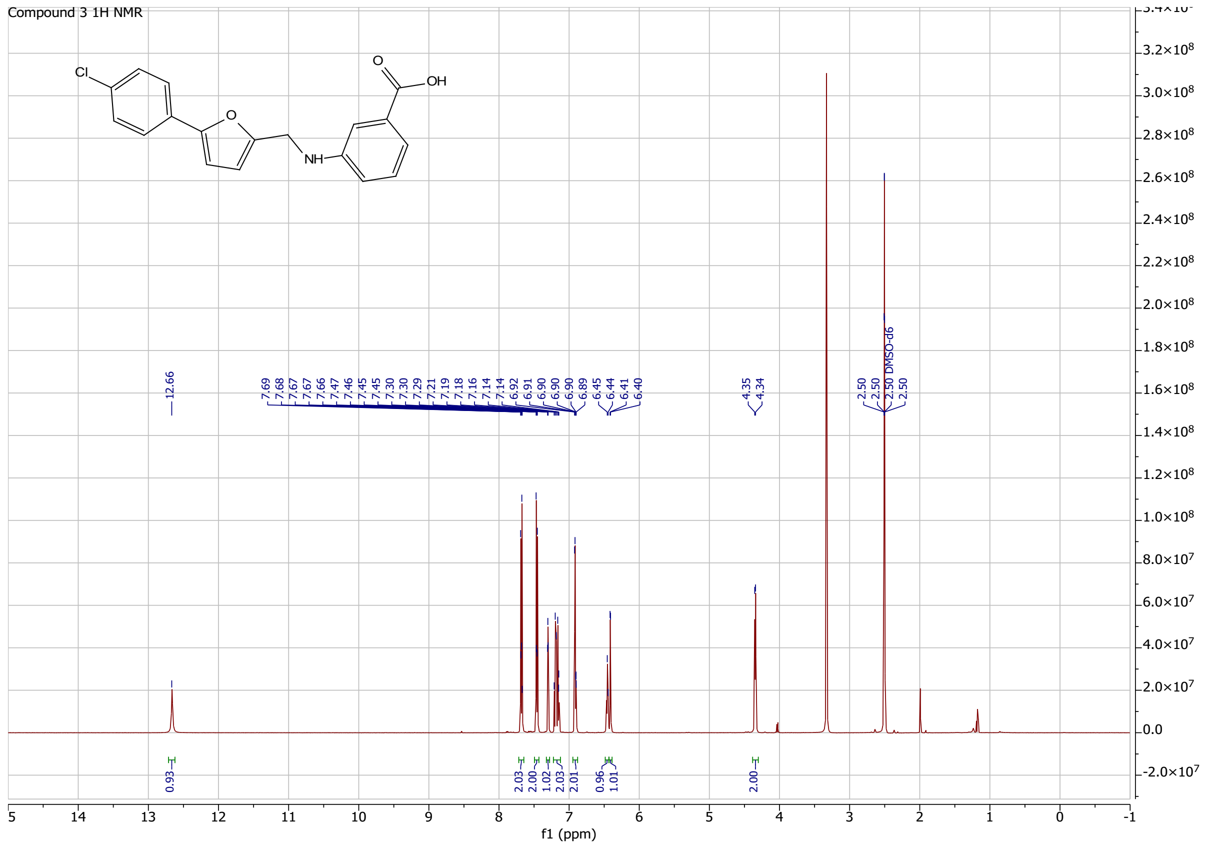

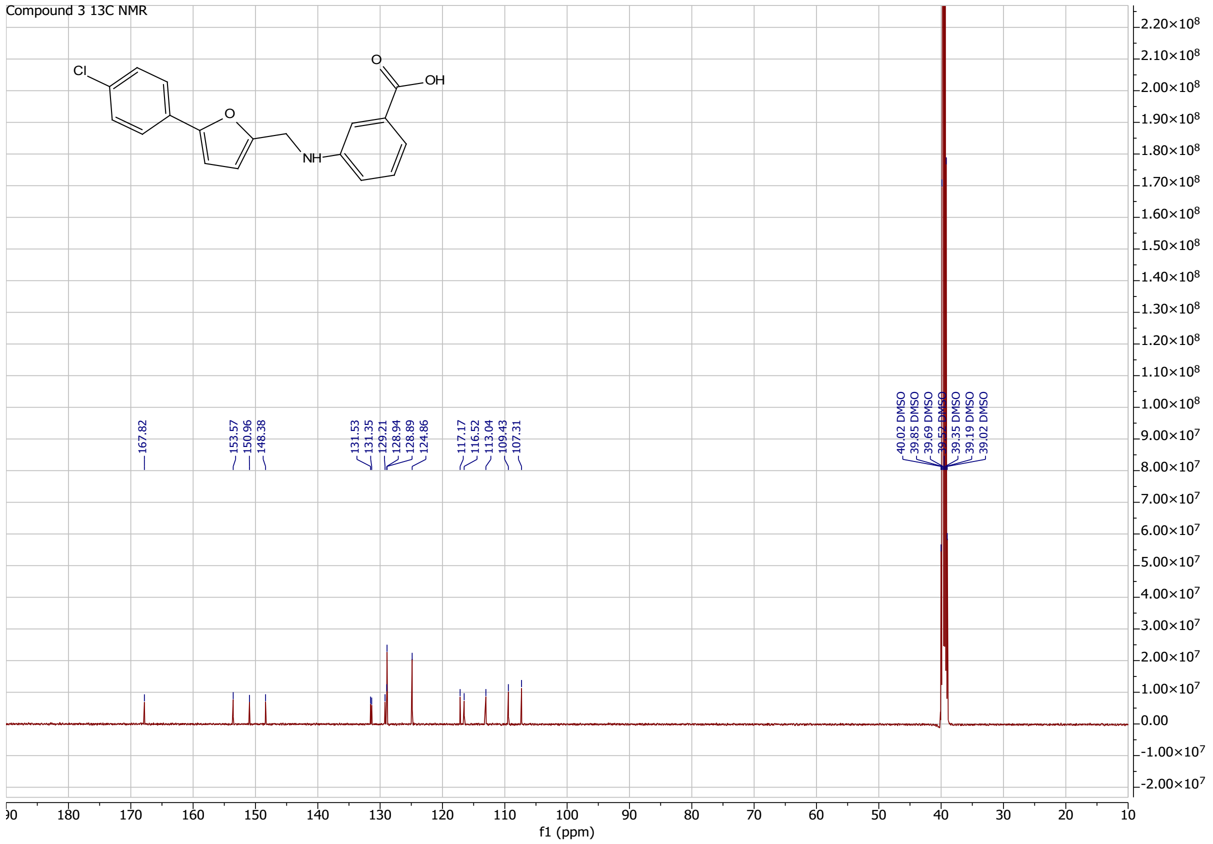
Compound 3 13C NMR
Cl
O
O
OH
NH
167.82
153.57
150.96
148.38
131.53
131.35
129.21
128.94
128.89
124.86
117.17
116.52
113.04
109.43
107.31
40.02 DMSO
39.85 DMSO
39.69 DMSO
39.52 DMSO
39.35 DMSO
39.19 DMSO
39.02 DMSO
f1 (ppm)

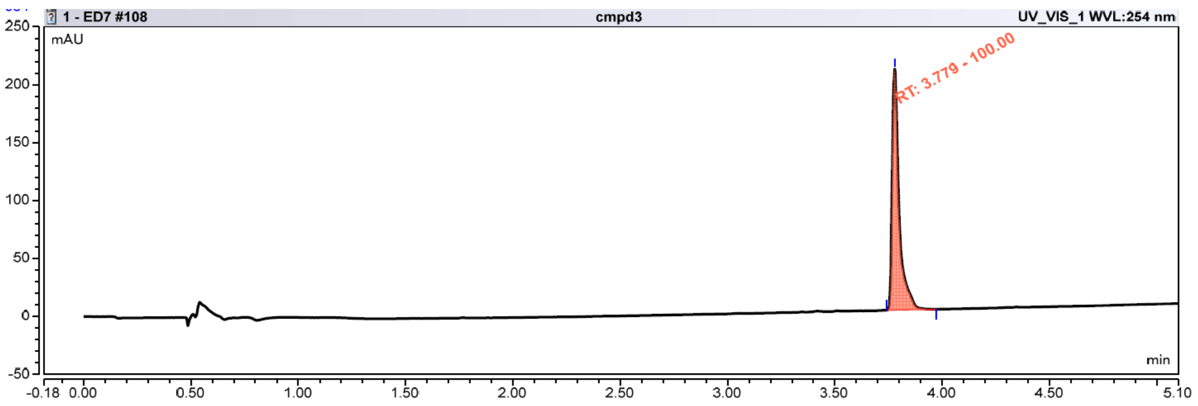
1 - ED7 #108
cmpd3
UV_VIS_1 WVL:254 nm
mAU
RT: 3.779 - 100.00
min

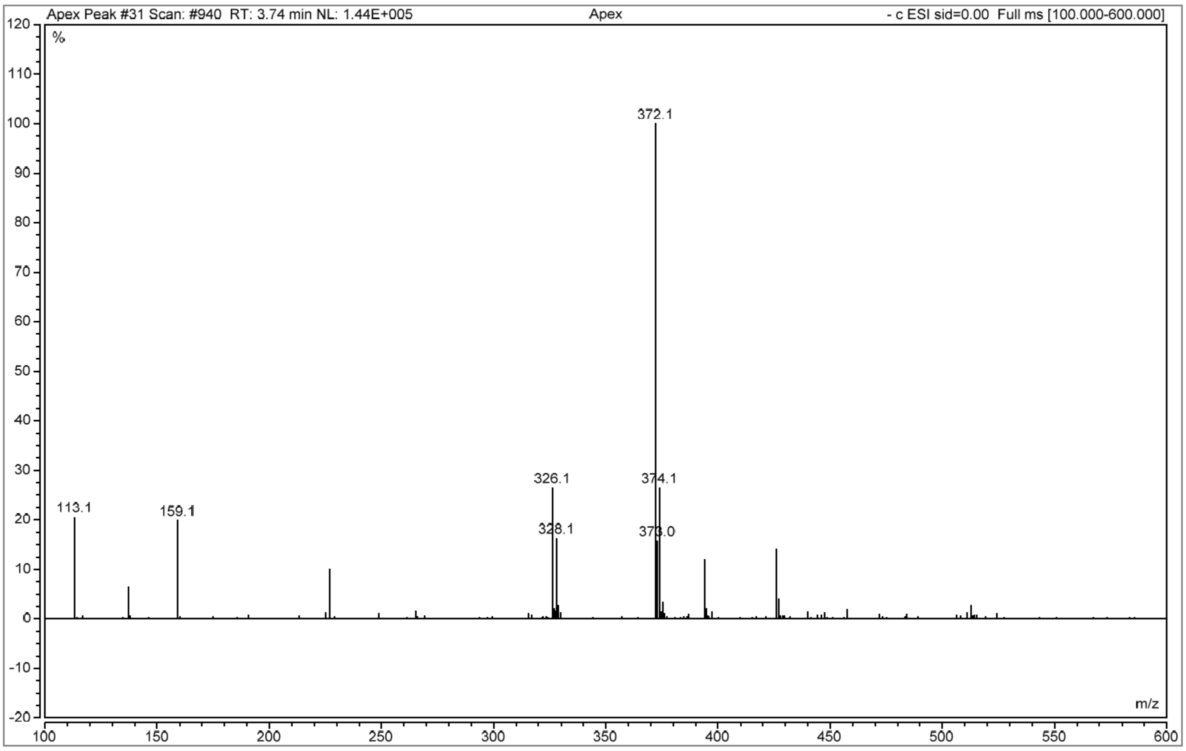

Figure S3. ^{1}H-, ^{13}C-NMR spectra of compound, UV chromatogram and mass spectra (LC-MS) of compound **3**.

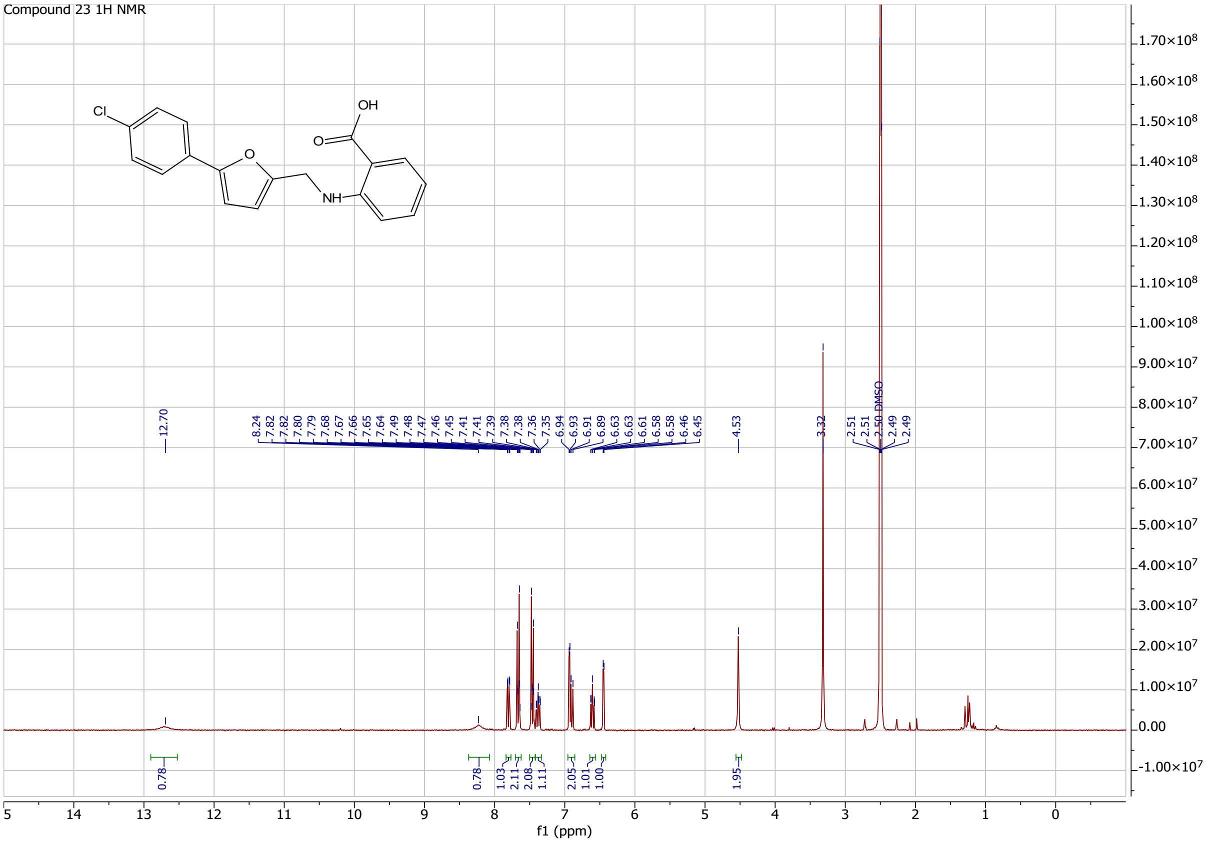

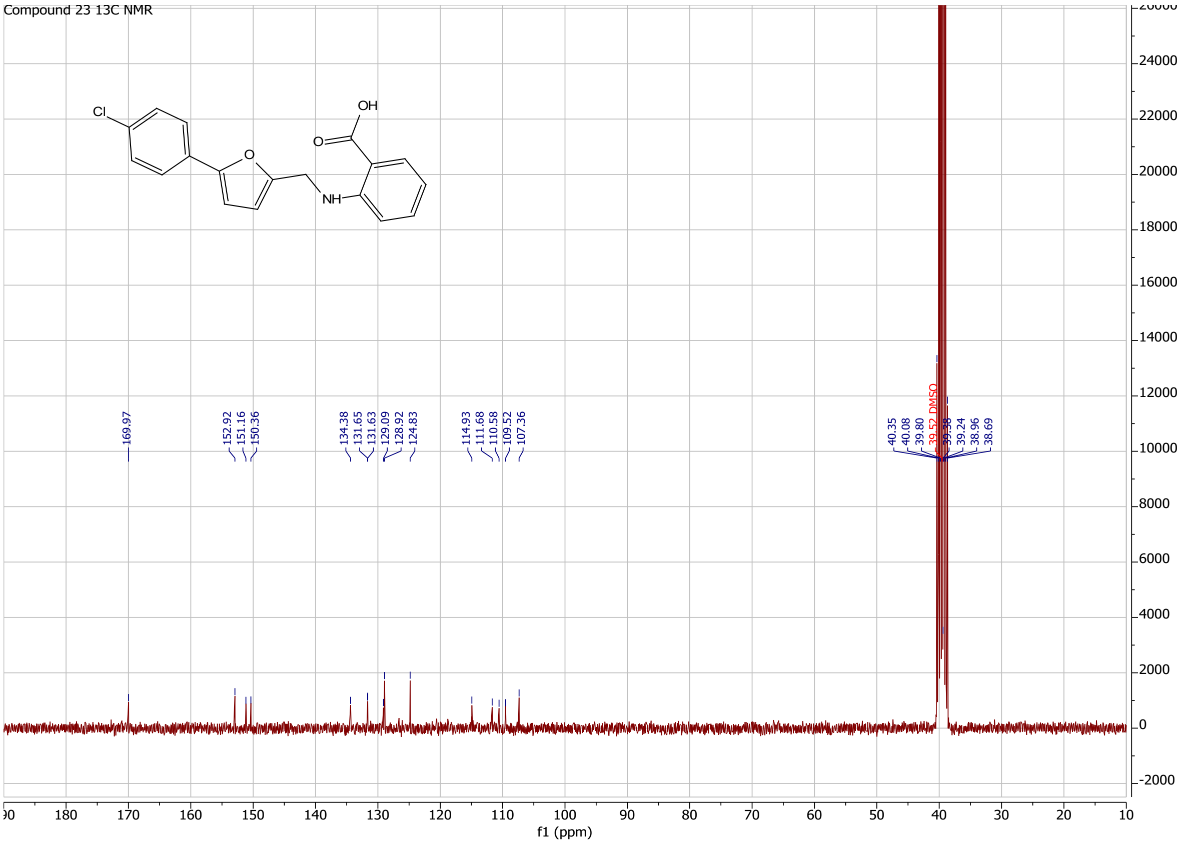
Compound 23 13C NMR
Cl
O
OH
O
NH
169.97
152.92
151.16
150.36
134.38
131.65
131.63
129.09
128.92
124.83
114.93
111.68
110.58
109.52
107.36
40.35
40.08
39.80
39.52 DMSO
39.24
38.96
38.69
f1 (ppm)

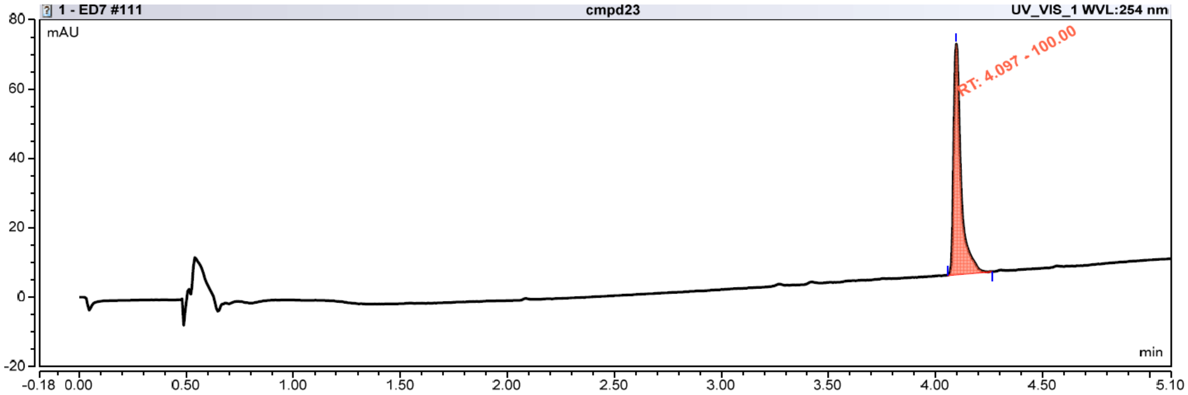
1 - ED7 #111
cmpd23
UV_VIS_1 WVL:254 nm
mAU
RT: 4.097 - 100.00
min

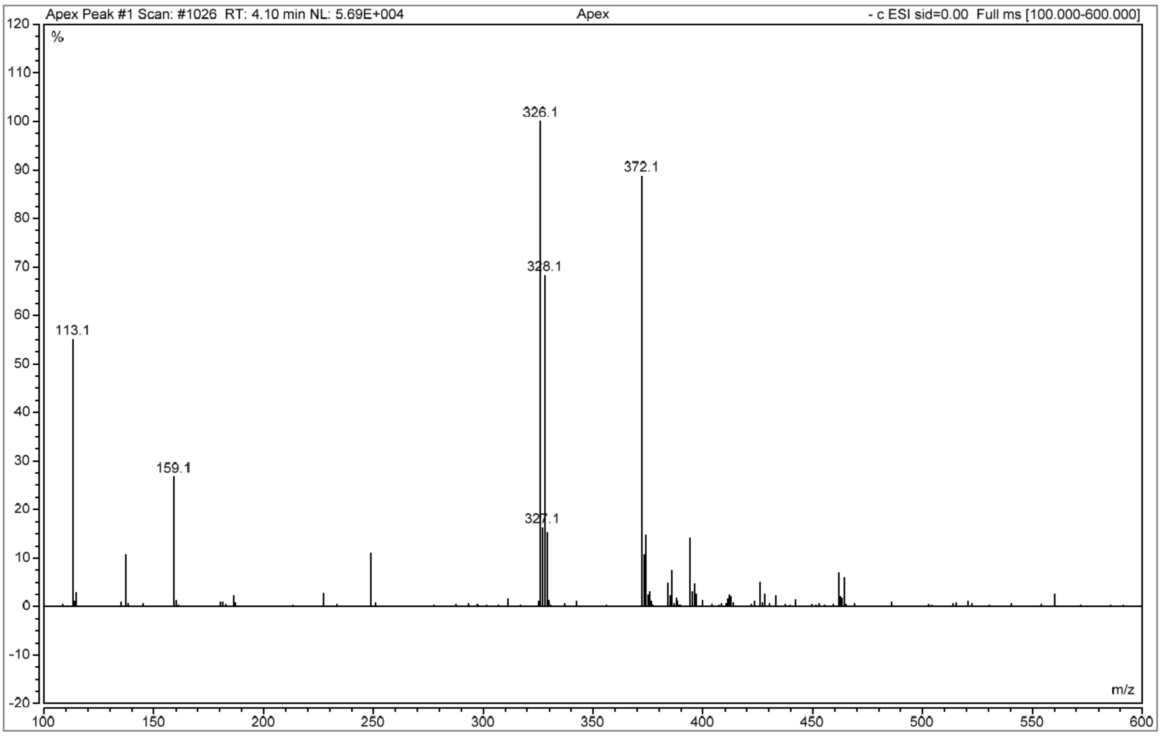

Figure S4. ^{1}H-, ^{13}C-NMR spectra, UV chromatogram and mass spectra (LC-MS) of compound **23**.

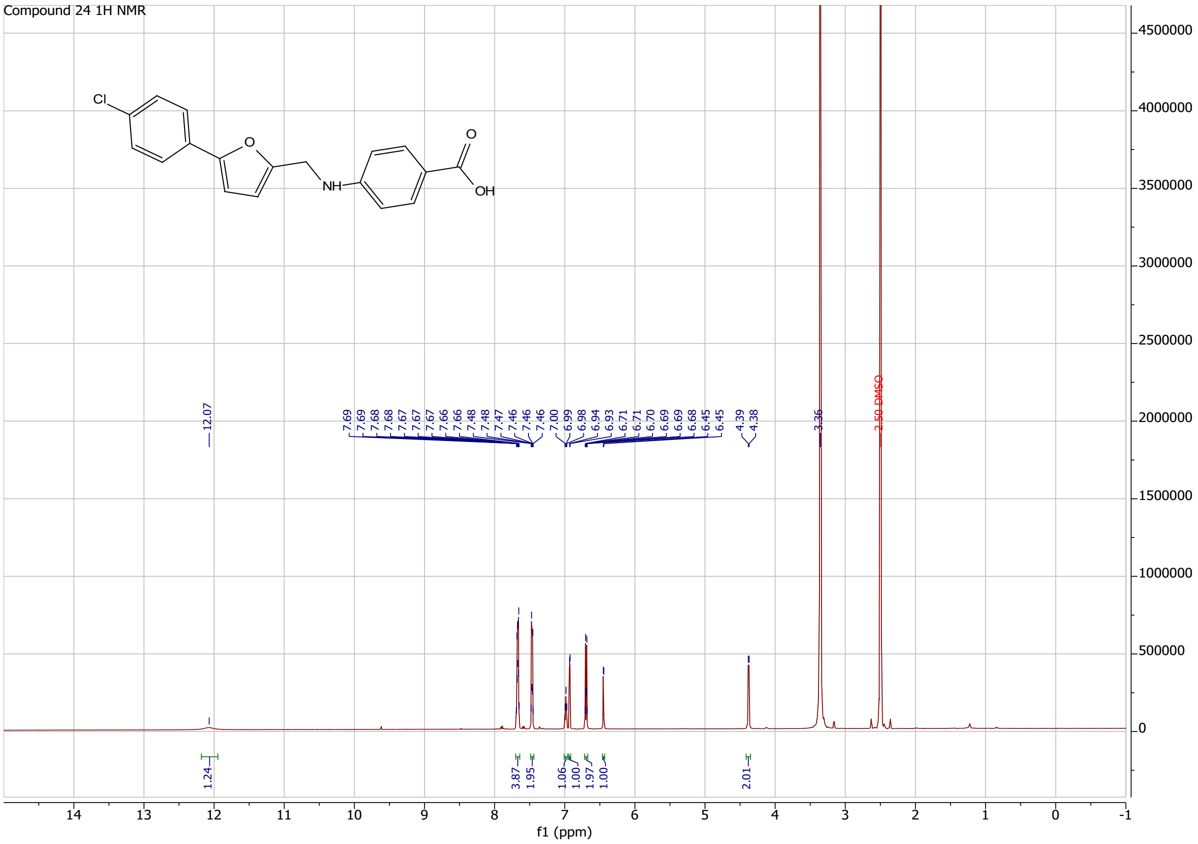
Compound 24 1H NMR
Cl
O
NH
O
OH
12.07
7.69
7.69
7.68
7.68
7.67
7.67
7.67
7.66
7.66
7.48
7.48
7.47
7.46
7.46
7.46
7.00
6.99
6.98
6.94
6.93
6.71
6.70
6.69
6.69
6.68
6.45
6.45
4.39
4.38
3.36
2.50 DMSO
1.24
3.87
1.95
1.06
1.00
1.97
1.00
2.01
f1 (ppm)

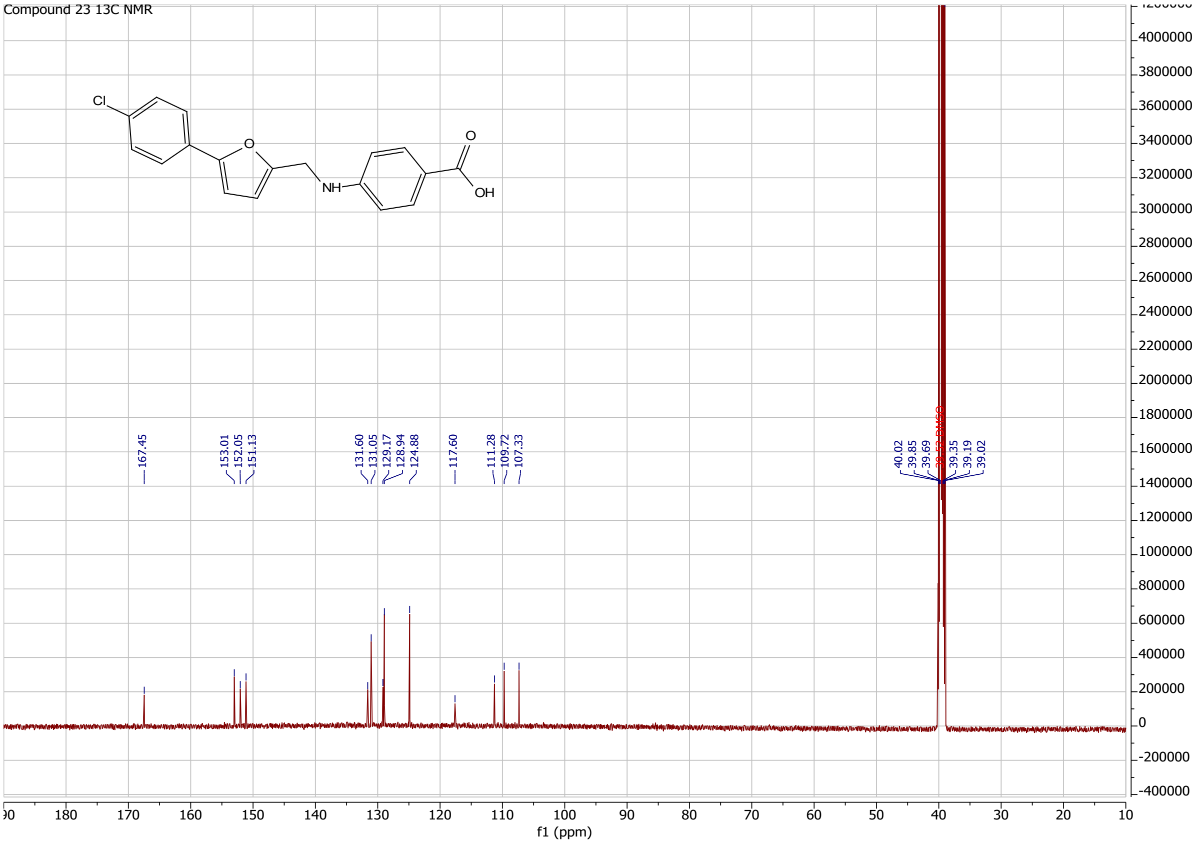
Compound 23 13C NMR
Cl
O
NH
O
OH
167.45
153.01
152.05
151.13
131.60
131.05
129.17
128.94
124.88
117.60
111.28
109.72
107.33
40.02
39.85
39.69
39.35
39.19
39.02
f1 (ppm)

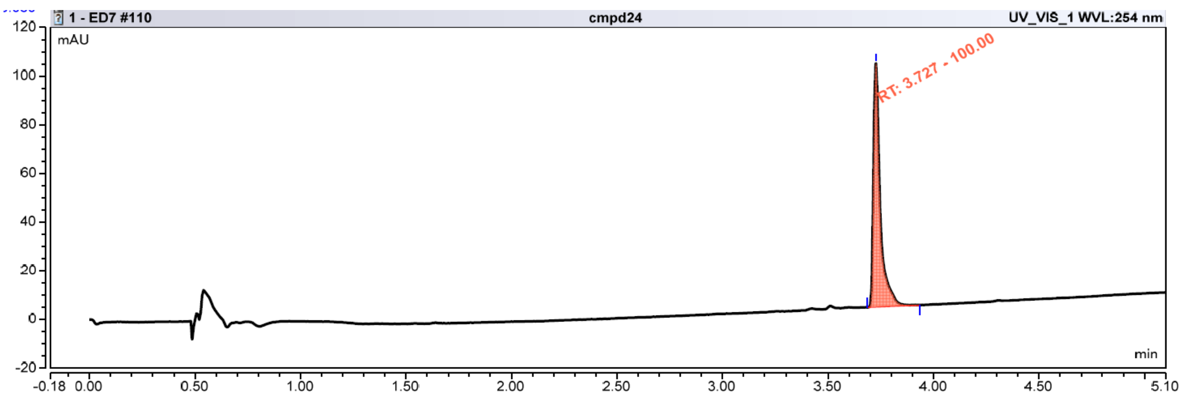

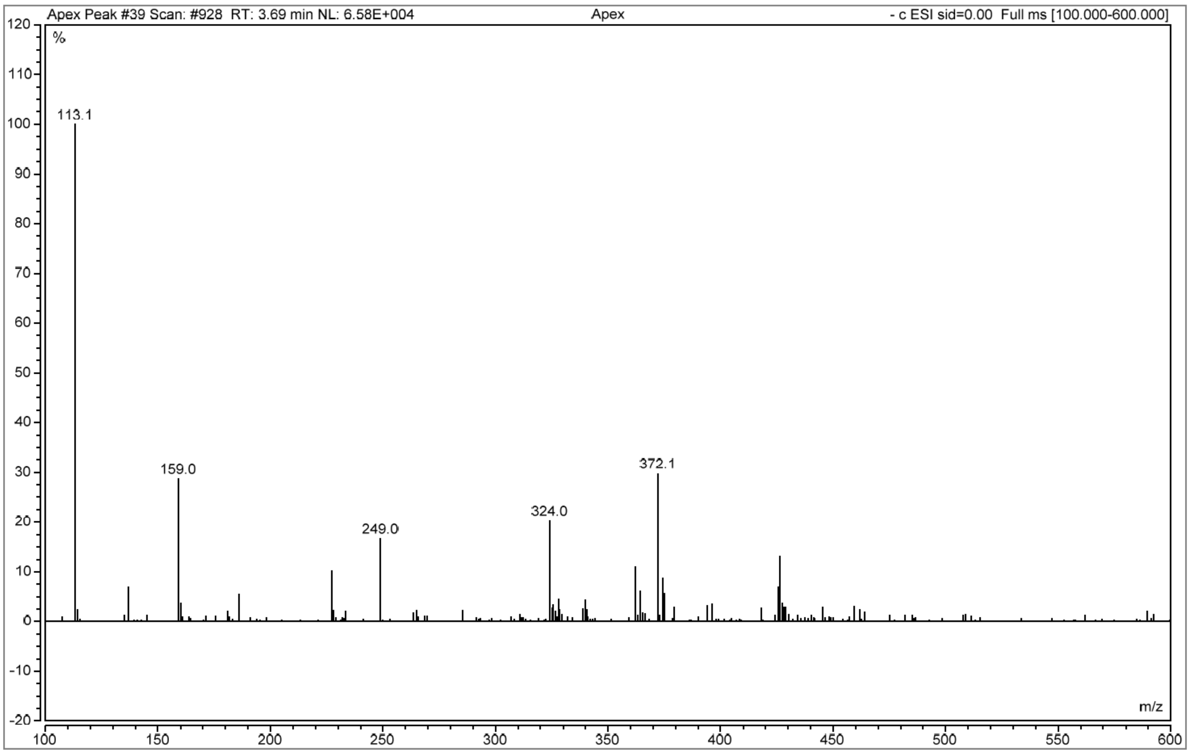

Figure S5. ^{1}H-, ^{13}C-NMR spectra, UV chromatogram and mass spectra (LC-MS) of compound **24**.

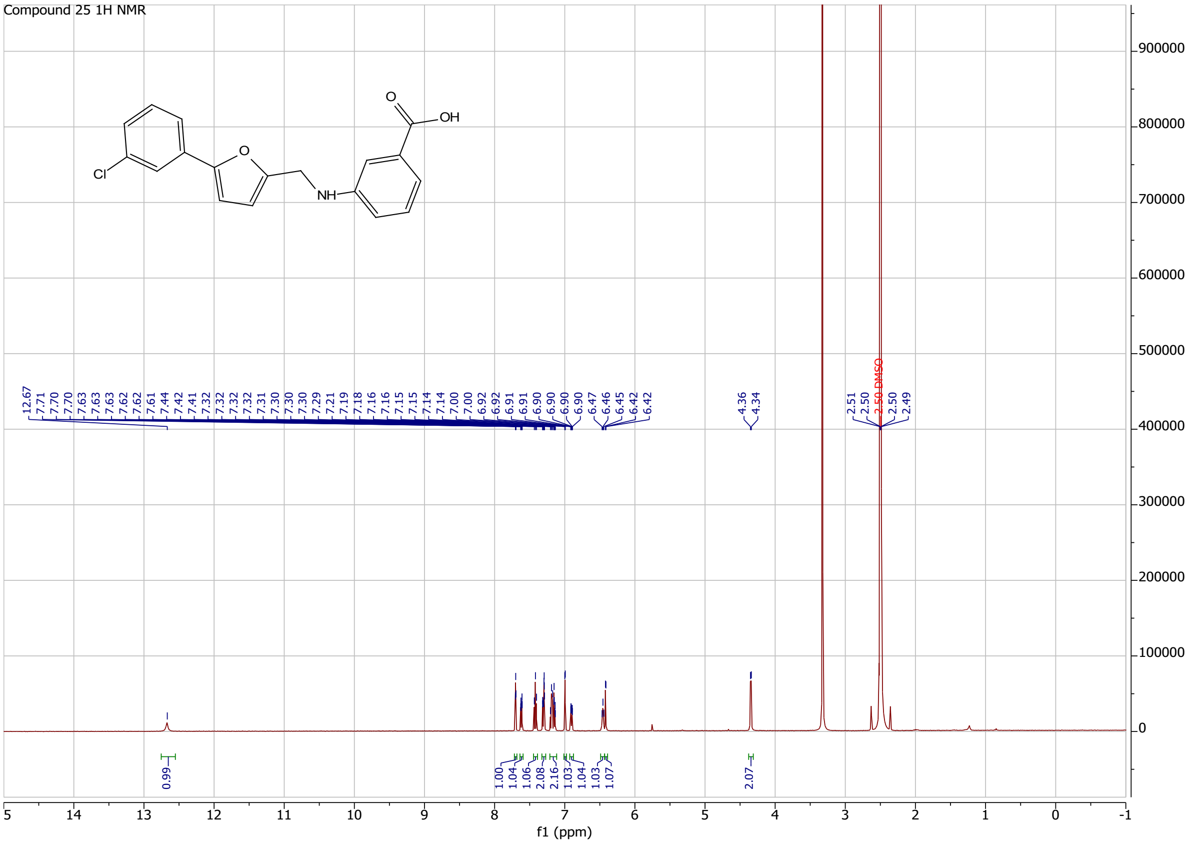
Compound 25 1H NMR
f1 (ppm)

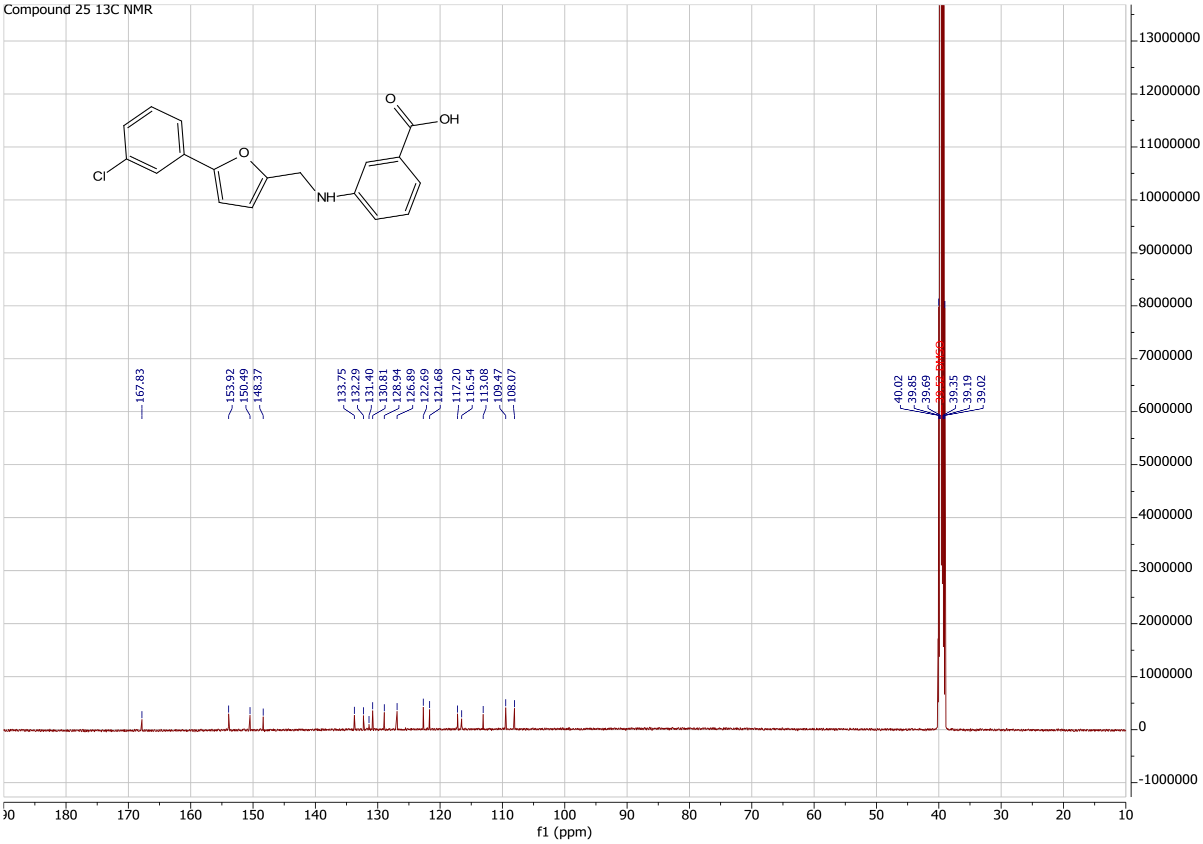
Compound 25 13C NMR
f1 (ppm)

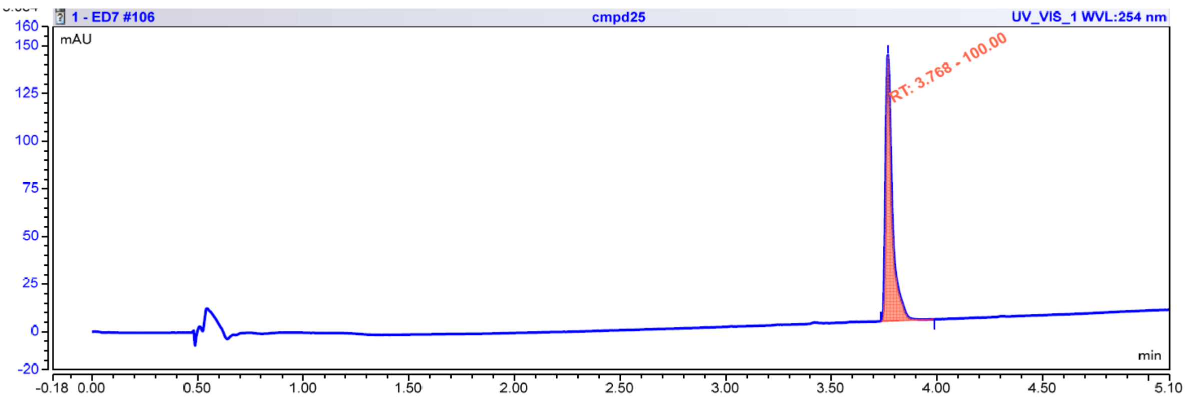

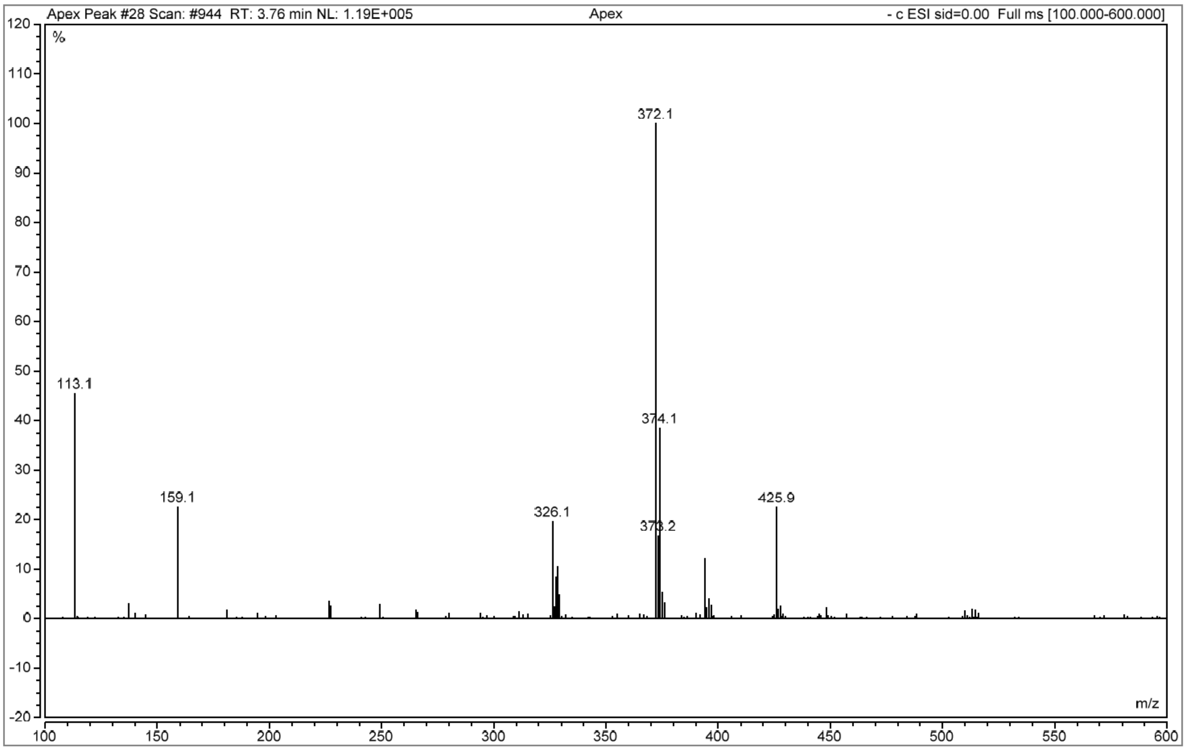

Figure S6. ^{1}H-, ^{13}C-NMR spectra, UV chromatogram and mass spectra (LC-MS) of compound **25**.

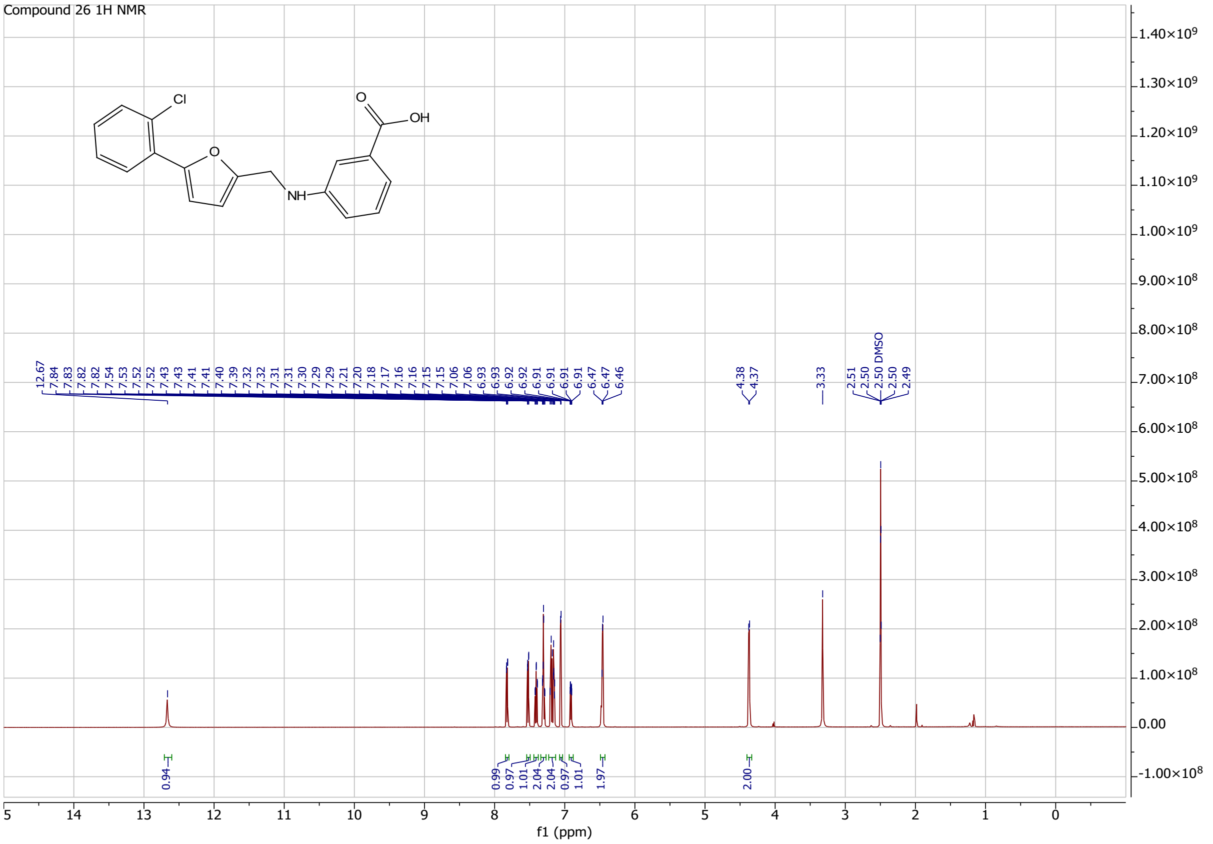
Compound 26 1H NMR
Cl
O
OH
O
NH
f1 (ppm)

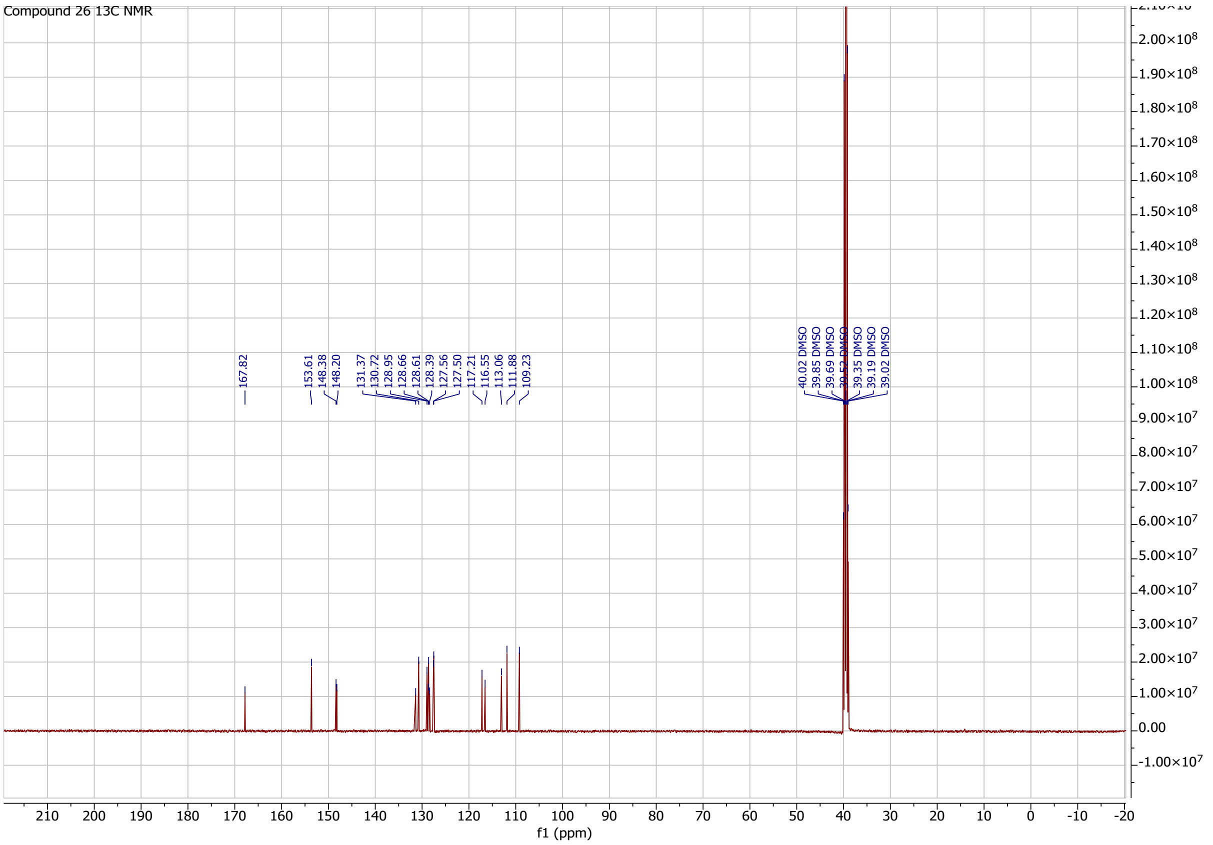
Compound 26 13C NMR
f1 (ppm)

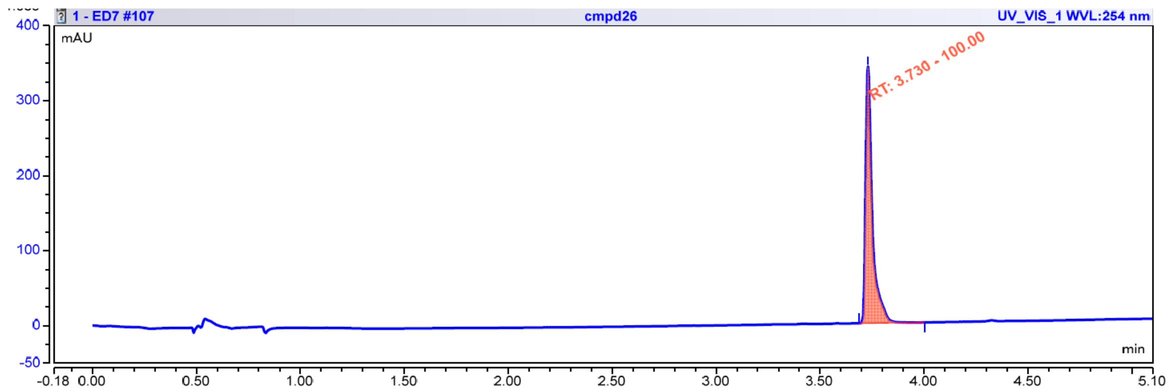

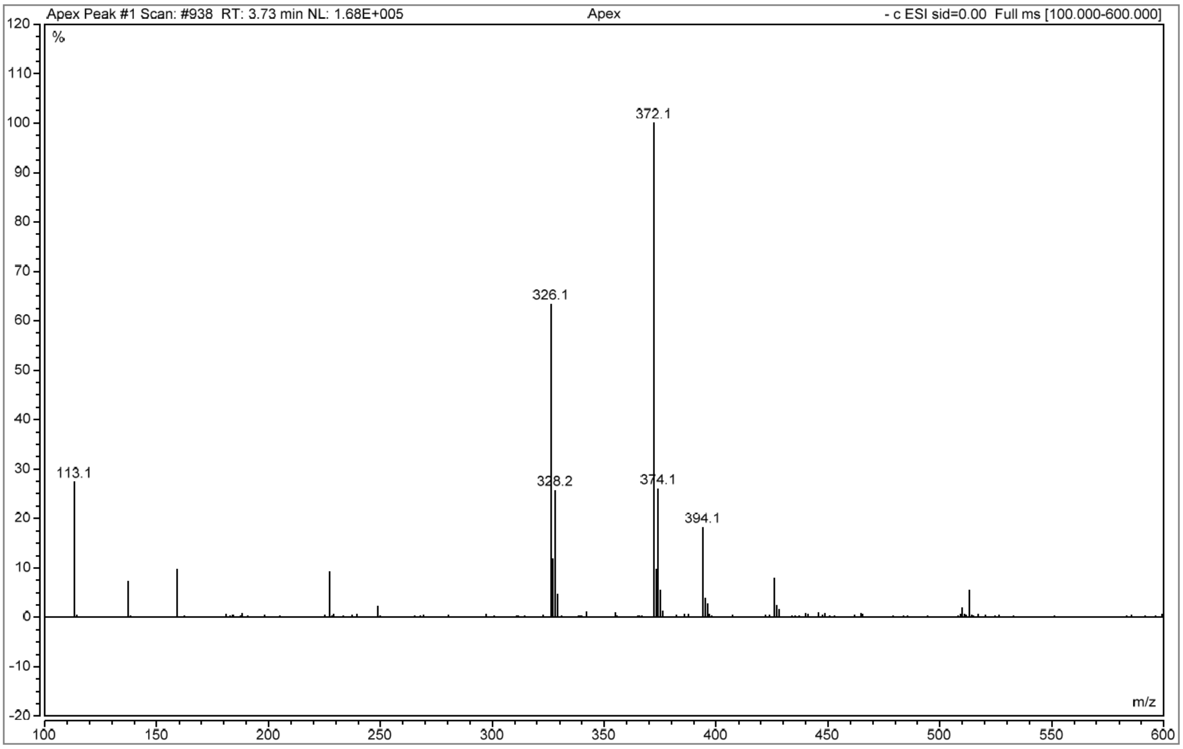

Figure S7. ^{1}H-, ^{13}C-NMR spectra, UV chromatogram and mass spectra (LC-MS) of compound **26**.

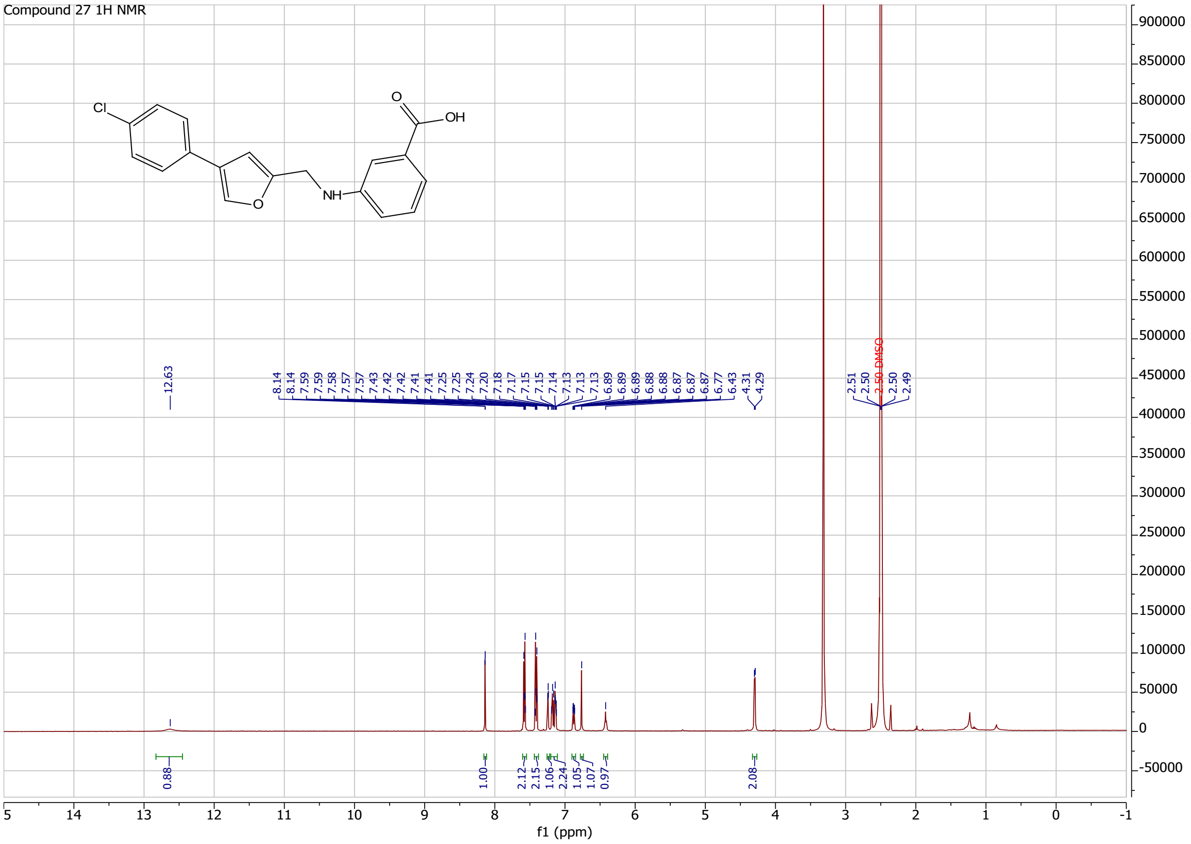
Compound 27 1H NMR
Cl
O
OH
NH
O
12.63
8.14 8.14 7.59 7.59 7.58 7.57 7.57 7.43 7.42 7.42 7.41 7.41 7.25 7.25 7.24 7.20 7.18 7.17 7.15 7.15 7.14 7.13 7.13 7.13 6.89 6.89 6.89 6.88 6.88 6.87 6.87 6.87 6.77 6.43 4.31 4.29
2.51 2.50 2.50 DMSO 2.50 2.49
0.88
1.00 2.12 2.15 1.06 2.24 1.05 1.07 0.97
2.08
f1 (ppm)

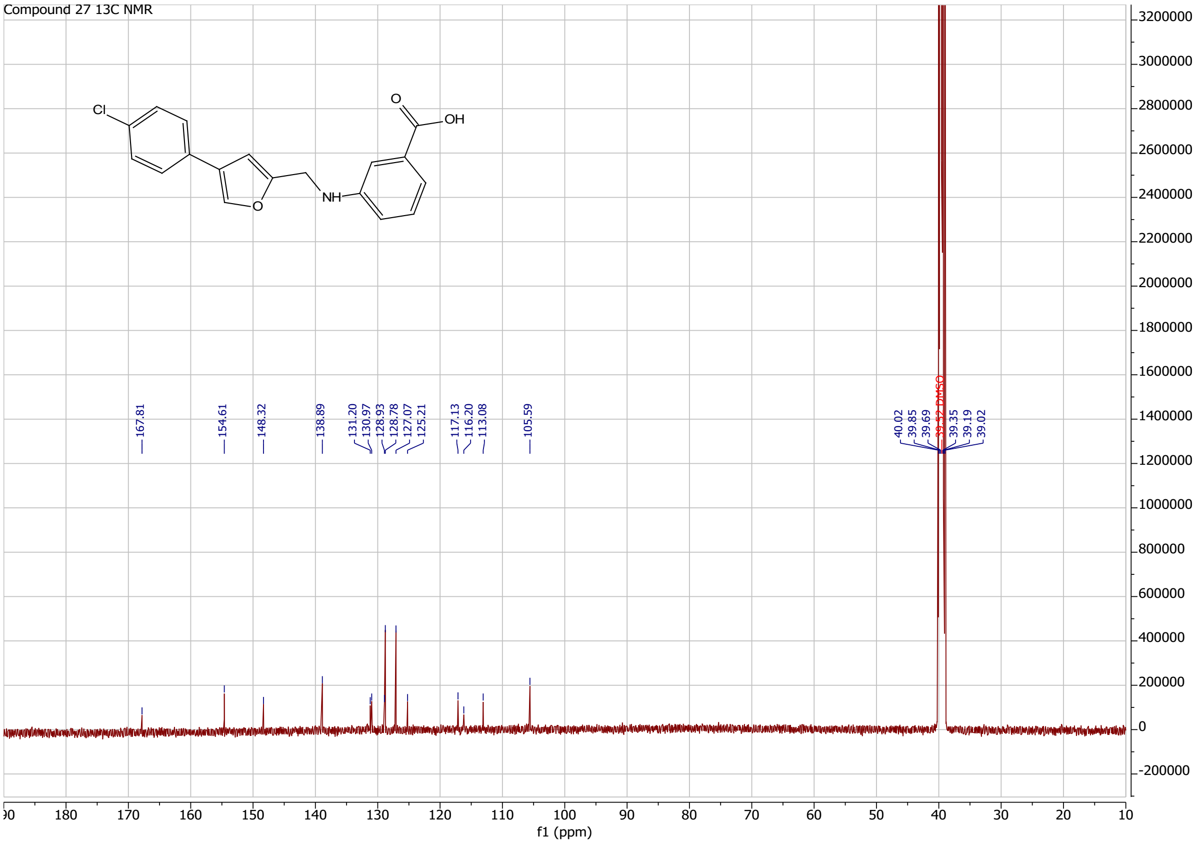
Compound 27 13C NMR
Cl
O
OH
NH
O
167.81
154.61
148.32
138.89
131.20 130.97 128.93 128.78 127.07 125.21
117.13 116.20 113.08
105.59
40.02 39.85 39.69 39.52 DMSO 39.35 39.19 39.02
f1 (ppm)

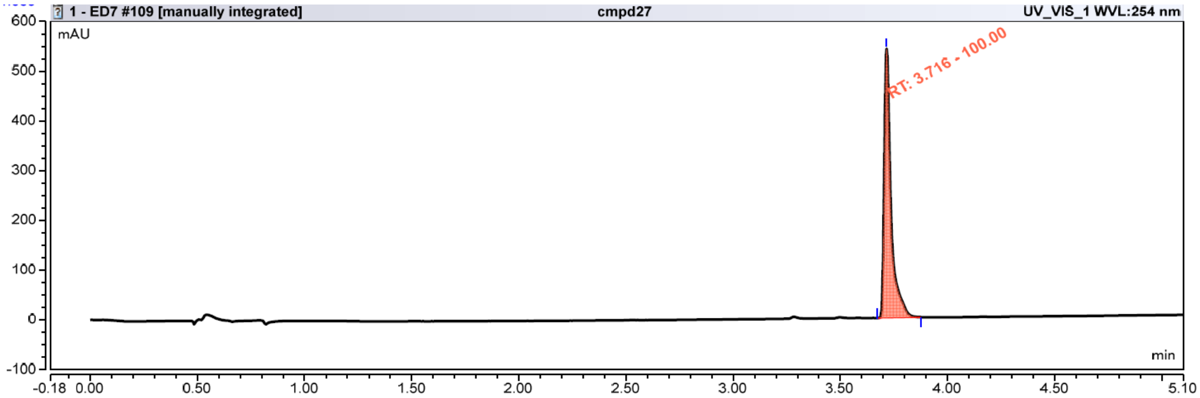

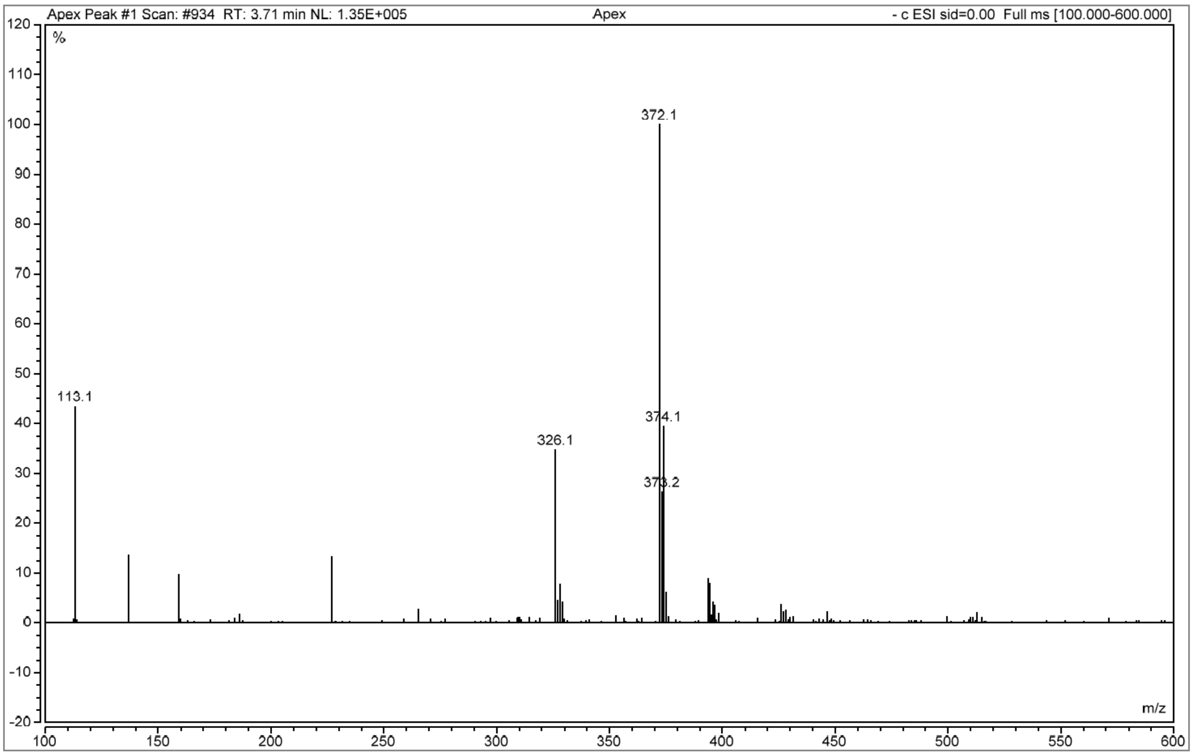

Figure S8. ^{1}H-, ^{13}C-NMR spectra, UV chromatogram and mass spectra (LC-MS) of compound **27**.

3. Biochemical assays

ECF-T inhibition assay

**Compound**	***Uptake into proteoliposome* assay** (*L. delbrueckii*)	***Bacterial uptake assay*** (*L. casei*)
**1**	IC_{50} = 282 ± 108 µM	IC_{50} = 315 ± 15 µM

Figure S9. Inhibition values of **1** against the uptake of radiolabeled folic acid into the proteoliposome and the bacterial assay. The uptake into proteoliposome assay is based on the ECF FolT2 transporter from *L. delbrueckii*, which is reconstituted in liposomes as reported elsewhere [1], while the bacterial uptake assay is based on *L. casei*.

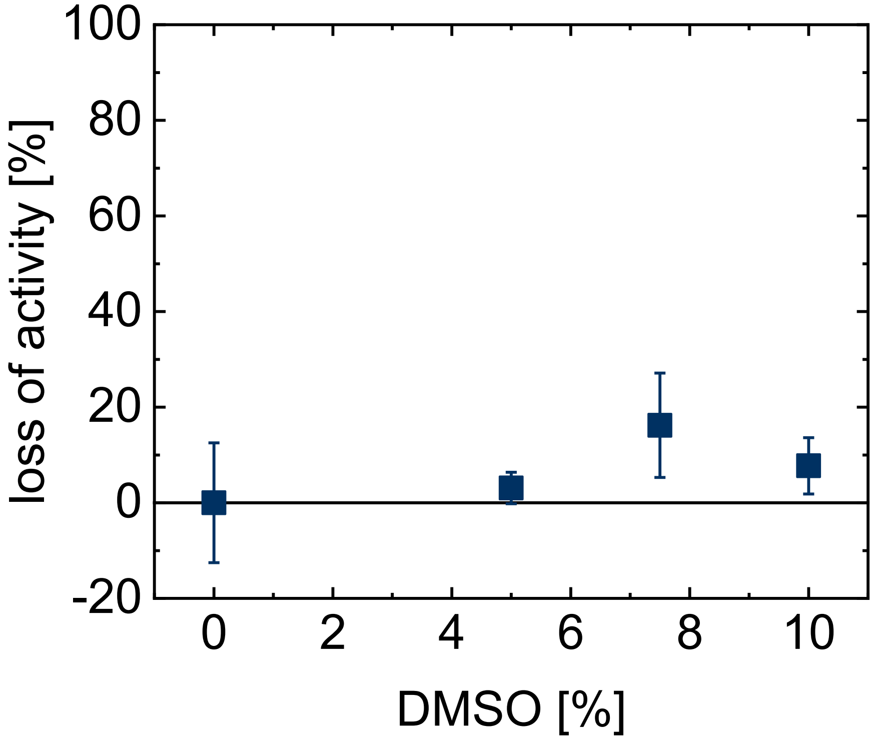

Figure S10. Loss of folate uptake activity in *L. casei* upon addition of different DMSO concentrations to the incubation mixture.

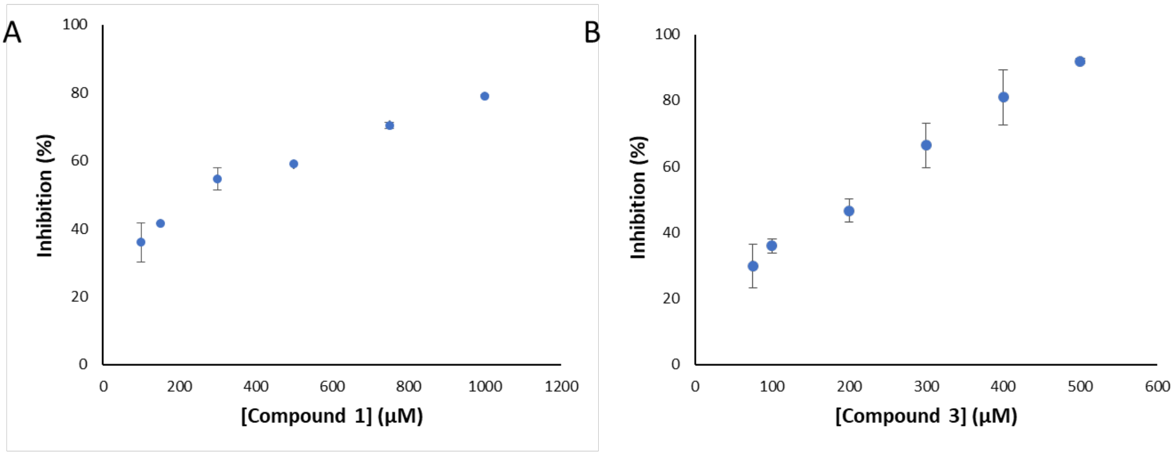

Figure S11. Determination of apparent IC_{50} values of compounds **1** (A) and **3** (B). The compound **1** and **3** exhibit an IC_{50} value of 315 ± 15 µM and 204 ± 22 µM, respectively. The errors in the graphs represent the standard deviation from two experiments.

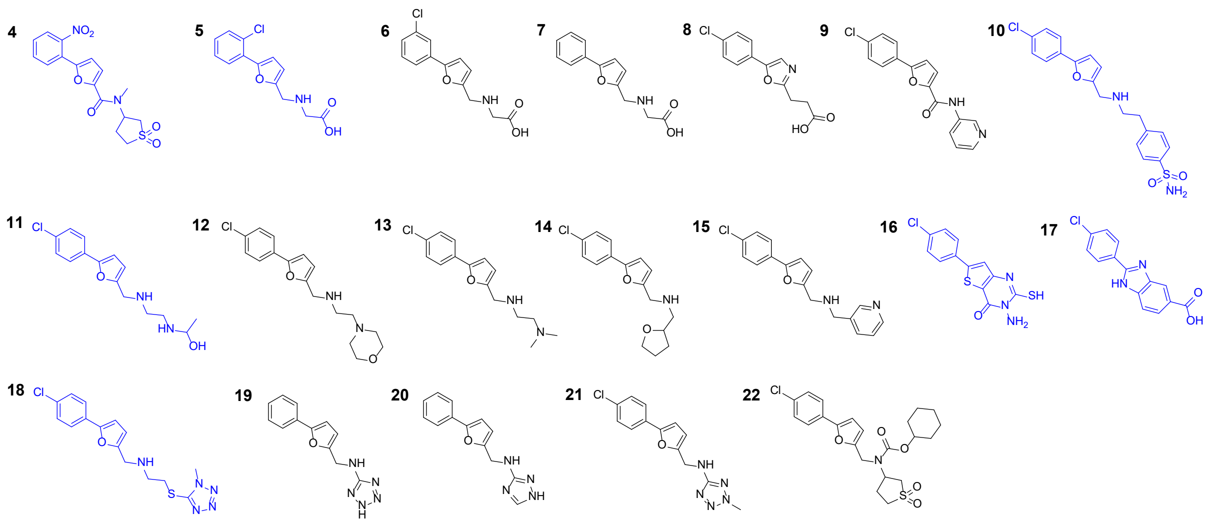

Figure S12. Screening a broad library (**4–22**) against ECF FolT2 transporter in the *L. casei* bacterial assay. Compounds colored in blue showed inhibition bigger than 20% when tested at 500 µM.

4. Antibacterial testing

Compounds were prepared as DMSO stocks and minimum inhibitory concentrations (MIC) were determined as described elsewhere. Bacteria were handled according to standard procedures and were obtained from the German Collection of Microorganisms and Cell Cultures (DSMZ) or were part of our internal strain collection. In brief, bacterial cultures were diluted in Tryptic Soy Broth (TSB; 1.7% peptone casein, 0.3% peptone soymeal, 0.25% glucose, 0.5% NaCl, 0.25% K_2HPO_4; pH 7.3) or Müller–Hinton broth (0.2% beef infusion solids, 1.75% casein hydrolysate, 0.15% starch, pH 7.4) to achieve a final inoculum of approximately 10^4 to 10^5 cfu/mL. Compounds were tested in serial dilution (0.06–128 µM) in 96-well plates and MIC values were determined by visual inspection after 16–20 h incubation at 37 °C.

5. Sequence conservation

Organisms	FolT	EcfT	EcfA1	EcfA2
Lactobacillus delbrueckii DSM 20081	Q1G929	A0A061BSU4	Q1GBJ0	Q1GBI9
Lactobacillus casei DSM 20011	S6BTZ8	S6BUV1	S6CK07	S6CA88

Figure S13: Accession numbers of the ECF FolT transporter (FolT, EcfT, EcfA1 &

EcfA2) of *L. delbrueckii* and *L. casei*. These codes were retrieved from the UniProtKB database [2].

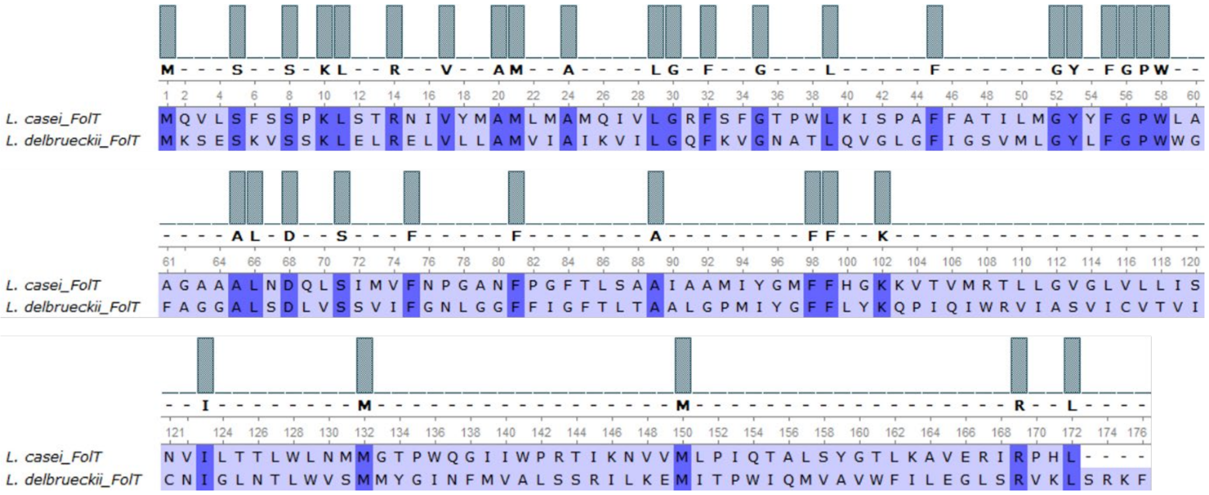

Figure S14: Sequence alignment of FolT membrane domain of *L. casei* (DSM 20011) and *L. delbrueckii* (DSM 20081). A color gradient from dark blue to white represents higher to lower conservation of amino acids between the various homologues. The residues with conservation ratio of 100% are colored in dark blue. The same color coding is used throughout the Supporting Information for figures of this type. The alignments presented here were prepared on the UniProtKB website [2] and the figures were produced using the software Unipro UGENE [3].

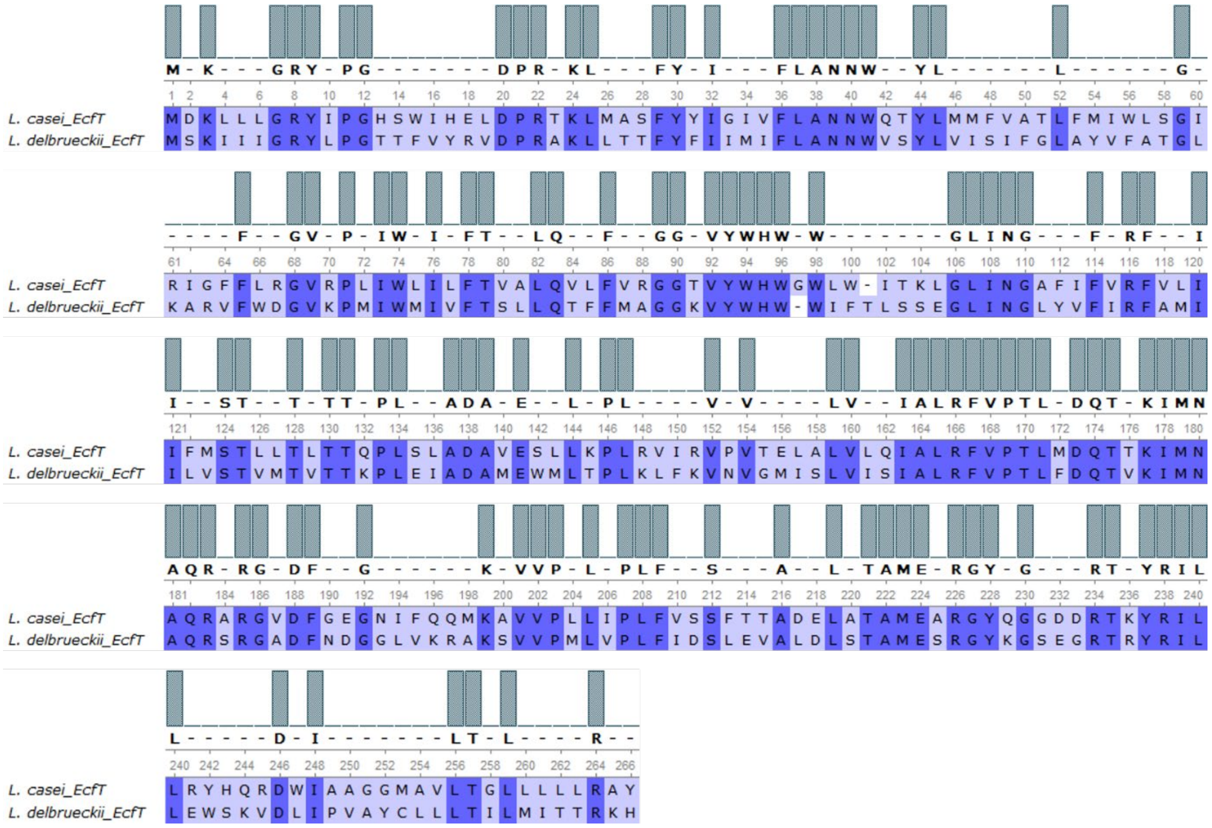

Figure S15: Sequence alignment of EcfT transmembrane domain of *L. casei* (DSM 20011) and *L. delbrueckii* (DSM 20081).

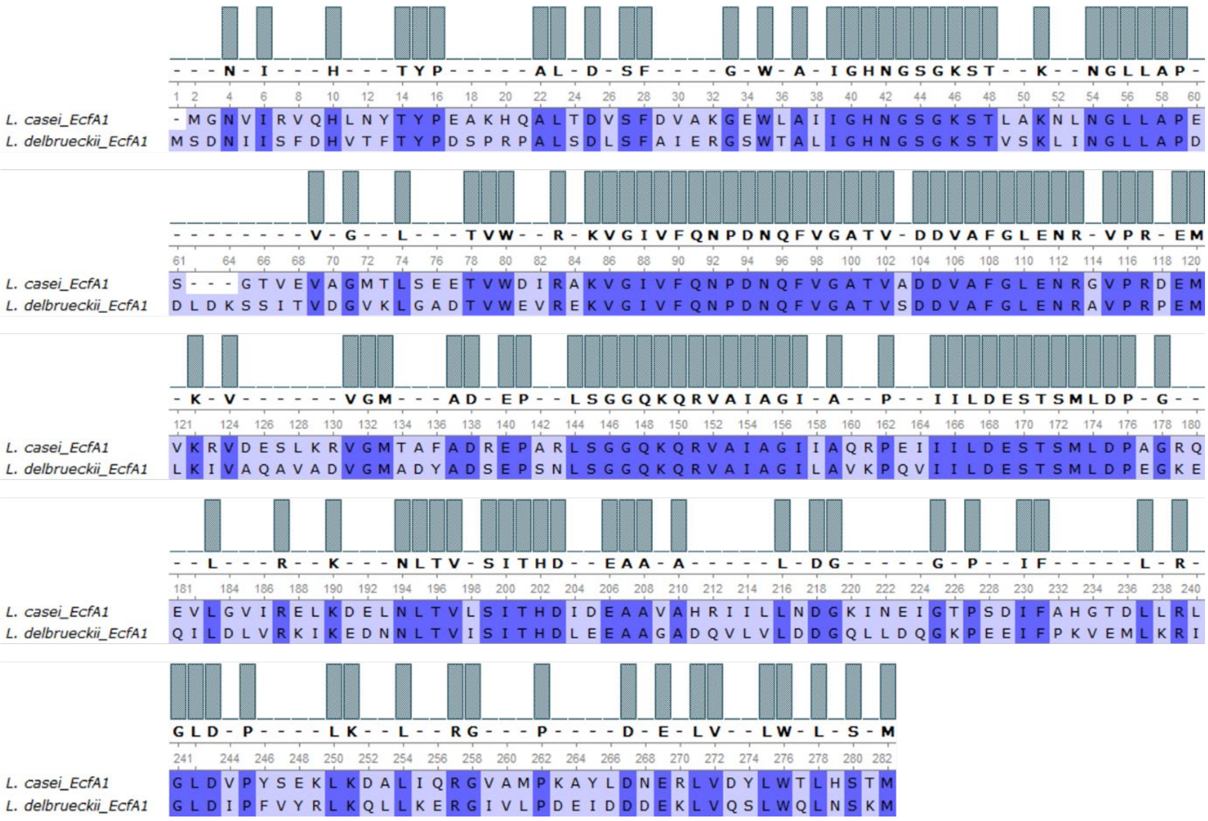

Figure S16: Sequence alignment of EcfA1 cytosolic domain of *L. casei* (DSM 20011) and *L. delbrueckii* (DSM 20081).

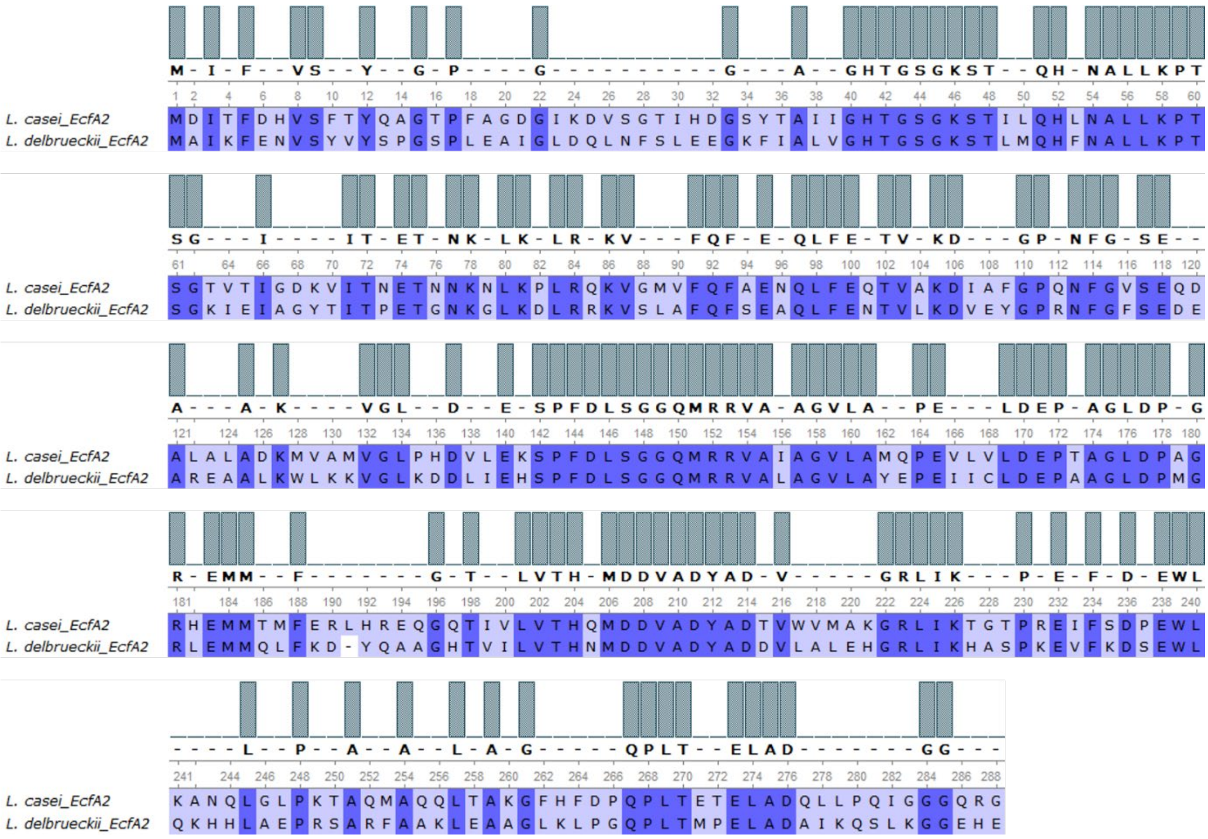

Figure S17: Sequence alignment of EcfA2 cytosolic domain of *L. casei* (DSM 20011) and *L. delbrueckii* (DSM 20081).

**Organisms (*L. Delbrueckii*)**	**FolT Identity (%)**	**EcfT Identity (%)**	**EcfA1 Identity (%)**	**EcfA2 Identity (%)**
L. casei	33	48	54	53

Figure S18: Sequence identity of the proteins that constitute the ECF_FolT transporter (FolT, EcfT, EcfA1 and EcfA2). The sequence of the proteins from *L. delbrueckii* are compared with those from *L. casei*.

6. References

1. Diamanti E., Setyawati I., Bousis S., Souza P.C.T., Mojas L., Swier L., Haupenthal J., Gibson P., Volz C., Stanek W., Jaeger M., Herrmann J., Marrink S., Veening J-W., Müller R., Slotboom D.J., Hirsch A.K.H., 10.26434/chemrxiv-2021-xq08b-v2.

2. UniProT, C. UniProt: the universal protein knowledgebase in 2021. *Nucleic Acids Research*, **2021**, 49, (D1), D480–D489.

3. Okonechnikov, K.; Golosova, O.; Fursov, M.; team, U., Unipro UGENE: a unified bioinformatics toolkit. *Bioinformatics* **2012,** *28* (8), 1166-7.
